# An estimated 400–800 million tons of prey are annually killed by the global spider community

**DOI:** 10.1007/s00114-017-1440-1

**Published:** 2017-03-14

**Authors:** Martin Nyffeler, Klaus Birkhofer

**Affiliations:** 1 Section of Conservation Biology, Department of Environmental Sciences, University of Basel, CH-4056 Basel, Switzerland; 2 Department of Biology, Lund University, SE-223 62 Lund, Sweden; 3 Chair of Ecology, Brandenburg University of Technology Cottbus-Senftenberg, 03046 Cottbus, Germany

**Keywords:** Araneae, Collembola, Insects, Global impact, Predation

## Abstract

Spiders have been suspected to be one of the most important groups of natural enemies of insects worldwide. To document the impact of the global spider community as insect predators, we present estimates of the biomass of annually killed insect prey. Our estimates assessed with two different methods suggest that the annual prey kill of the global spider community is in the range of 400–800 million metric tons (fresh weight), with insects and collembolans composing >90% of the captured prey. This equals approximately 1‰ of the global terrestrial net primary production. Spiders associated with forests and grasslands account for >95% of the annual prey kill of the global spider community, whereas spiders in other habitats are rather insignificant contributors over a full year. The spider communities associated with annual crops contribute less than 2% to the global annual prey kill. This, however, can be partly explained by the fact that annual crop fields are “disturbed habitats” with a low buildup of spider biomass and that agrobiont spiders often only kill prey over short time periods in a year. Our estimates are supported by the published results of exclusion experiments, showing that the number of herbivorous/detritivorous insects and collembolans increased significantly after spider removal from experimental plots. The presented estimates of the global annual prey kill and the relative contribution of spider predation in different biomes improve the general understanding of spider ecology and provide a first assessment of the global impact of this very important predator group.

## Introduction

Spiders, which evolved from an arachnid ancestor during the Devonian period around 400 million years ago, are among the most common and abundant predators in terrestrial ecosystems (Turnbull 1973; Coddington and Levi 1991; Selden et al. 1991). For instance, Turnbull (1973) calculated an overall mean density of 131 spiders m^−2^ based on assessments from many different areas of the globe, and Nyffeler (2000) found an overall mean density of 152 spiders m^−2^ for a large variety of grassland habitats. Under favorable conditions, spiders can reach peak densities of up to 1000 individuals m^−2^ (Ellenberg et al. 1986). At the present time, >45,000 species of spiders are described and those exhibit a very diverse range of lifestyles and foraging behaviors (Wise 1993; Platnick 2014). Barth (1997) partially attributes the evolutionary success of spiders to the fact that they are equipped with highly developed sensory systems providing individuals with detailed information about potential predators and prey in their surroundings. All spiders are carnivores, feeding predominantly on insects/collembolans and to a lesser extent on other spiders (Nyffeler 1999; Birkhofer and Wolters 2012; Pekár and Toft 2015). Very rarely nonarthropod prey and sometimes even plant materials are consumed as a supplement to the arthropod diet (Symondson et al. 2002; Foelix 2011; Nyffeler et al. 2016). Spiders have efficient survival mechanisms given that they are so numerous and widespread. Their capability to survive under extreme conditions and to disperse by ballooning through the atmosphere from place to place on silken threads allowed spiders to colonize a wide variety of different terrestrial habitats. Some spiders can travel distances of up to 30 km in a single day (Thomas et al. 2003). There is hardly any terrestrial area on this globe where spiders would be missing. “….Spiders exist in the most northern islands of the Arctic, the hottest and most arid of deserts, at the highest altitudes of any living organisms, in the depths of caves, in the intertidal zone of ocean shores, in bogs and ponds, on high, arid moorlands, sand dunes, and flood plains” (Turnbull 1973). Because of their high abundance and predominantly insectivorous feeding habits, spiders are suspected to be the main predators of insects (Selden 2016).

Due to their secretive lifestyle—some species are for example nocturnal or hunt in litter and soil habitats—the predatory activities of the spiders remain largely unnoticed and it is therefore difficult to estimate their impact on prey. To illustrate the impact of spiders as insect predators, two arachnologists—W. S. Bristowe from England and A. L. Turnbull from Canada—previously tried to quantify the food consumption of spiders by means of extrapolations. In his work “A Book of Spiders,” Bristowe (1947) estimated that England and Wales are populated by roughly 2.2 × 10^12^ spiders and that these spiders may kill ≈2.2 × 10^14^ insects annually. Bristowe (1958) went one step further claiming that the weight of insects consumed by the entire British spider fauna would exceed the combined weight of all the humans in Great Britain. Nyffeler (2000) conducted a recalculation of Bristowe’s estimate and came to the conclusion that Bristowe probably overestimated the overall prey kill of the spiders since Great Britain consists predominantly of agricultural land characterized by reduced annual prey consumption. Turnbull (1973), on the other hand, estimated that the average total weight of food annually consumed by spiders would amount to 4.25 × 10^3^ metric tons km^−2^ land area. This latter value is of the same magnitude as the net primary production in terrestrial ecosystems, which is irreconcilable with ecological theory (Nyffeler 2000).

Here, we provide estimates for the standing biomass of the global spider community and the annual biomass of prey that is killed by the global spider community in individual biome types and worldwide based on literature data.

## Methods

### Estimate of the standing biomass of the global spider community

A total of 65 values of spider biomass m^−2^ were gathered from the literature. The data were assigned to the following seven groups of terrestrial biomes: (1) tropical forests, (2) temperate and boreal forests, (3) tropical grasslands and savannas, (4) temperate grasslands (incl. old fields, permanent pastures, mown meadows) and Mediterranean shrublands, (5) annual cropland, (6) deserts, and (7) Arctic tundra. To retrieve comparable data, all values were converted to fresh weight m^−2^ taking into account an average water content of the spider body of ≈75% (Pulz 1987). The data were pooled by computing an average biomass value (g m^−2^) for each biome type. By extrapolation—using the global land cover data from Saugier et al. (2001)—the standing biomass of the global spider community was then computed (Table 1).

**Table 1:** Estimated standing biomass of the global spider community based on grams per square meter values ($\overline{\mathrm{x}}$ ± SE, all values expressed as fresh weight)

Biome type	Number of assessments	$\overline{\mathrm{x}}$ Biomass (g m^-2^)	Area in m^2^	Biomass subtotal (g)
		(*B*)	(*Y*)	(*B*) × (*Y*)
Tropical forests^a^	7	0.38 ± 0.147	17.5 × 10^12^	6.65 × 10^12^
Temperate and boreal forests^b^	18	0.40 ± 0.054	24.1 × 10^12^	9.64 × 10^12^
Tropical grasslands and savannas^c^	11	0.18 ± 0.046	27.6 × 10^12^	4.97 × 10^12^
Temperate grasslands and Mediterranean shrublands^d^	8	0.16 ± 0.013	17.8 × 10^12^	2.85 × 10^12^
Annual cropland^e^	13	0.017 ± 0.004	13.5 × 10^12^	0.23 × 10^12^
Deserts^f^	3	0.020 ± 0.006	27.7 × 10^12^	0.55 × 10^12^
Arctic tundra^g^	5	0.035 ± 0.009	5.6 × 10^12^	0.20 × 10^12^
Global total (without ice-covered area)			133.8 × 10^12^	25.09 × 10^12^

^a^Anichkin et al. (2007); Raub and Höfer (2010); Göltenboth et al. (2006)

^b^van der Drift (1951); Kitazawa (1967); Gist and Crossley (1975); Huhta and Koskenniemi (1975); Luczak (1975); Miller and Obrtel (1975); Persson et al. (1980); Axelsson et al. (1984); Meyer et al. (1984); Ellenberg et al. (1986); Niijima (1998); Huhta (2002); Scheu et al. (2003)

^c^Gillon and Gillon (1967); Malaisse and Benoit (1979); Decaëns et al. (2001)

^d^Stöckli (1950); Cherrett (1964); Delchev and Kajak (1974); Persson and Lohm (1977); Hutchinson and King (1980); Curry (1986)

^e^Basedow et al. (1991); Basedow (1993); Blumberg et al. (1997); Decaëns et al. (2001); Nyffeler and Sunderland (2003)

^f^Chew (1961); Mispagel and Sleeper (1983); combined data Polis (1991)/Boulton and Polis (1999)

^g^Petersen and Luxton (1982); Byzova et al. (1995)

### Estimate of the annual prey kill by the global spider community

We used simple models involving few assumptions as is advised in cases where a field of study is still largely undeveloped (Weathers and Weathers 1983). Two different approaches were taken to estimate the annual prey kill of the global spider community. In the case of method I, the estimate is based on the spiders’ food requirements per unit body weight known from the literature in combination with spider biomass m^−2^ values (data for various biome types being taken from the literature), whereas method II is based on complete assessments of the spiders’ annual prey kill (e.g., prey censuses in the field combined with web density estimates) in selected biome types published in the literature. The two estimation methods are based on different sets of studies (with zero overlap of data between the two methods).

Prey items that are killed in webs but remain uneaten are considered prey as well (Nentwig 1987). This issue is playing a role when dealing with spider communities dominated by large-sized orb-weaving spiders known to often kill prey in excess (“wasteful killing”). Accordingly, authors who conducted prey censuses of large-sized orb weavers usually have taken into account the “prey killed in webs but uneaten” in their assessments (see Robinson and Robinson 1974; Kajak et al. 1971; Nyffeler 1976, 1982; Nyffeler and Benz 1978, 1979, 1989; Malt 1996). In other studies dealing predominantly with cursorial hunters and/or small-sized web-building spiders which rarely catch prey in excess under natural conditions (Nyffeler, pers. obs.), this issue was disregarded.

*Method I* **:** Based on the spider biomass m^−2^ values from Table 1 and an assumptive food intake rate (mg prey per mg spider body mass day^−1^), the prey kill m^−2^ day^−1^ of spider communities for each of the seven biome types was computed. These values were extrapolated to prey kill m^−2^ year^−1^ for each biome type, considering the length of the spiders’ feeding season (in days, see assumptions below). By multiplying the prey kill m^−2^ year^−1^ with the corresponding area size of a particular biome type (based on Saugier et al. 2001), a prey kill subtotal was derived for each biome type. Summing up the seven subtotals produced an estimate of the annual prey kill of the global spider community (Table 2). The estimate derived by method I was based on the following assumptions:

**Table 2:** Estimated annual prey kill (fresh weight) of the global spider community assessed with method I

Biome type	Number of assessments	Prey kill (g m^−2^ year^−1^)	Area in m^2^	Prey kill of entire area (g year^−1^)
		(*X*)	(*Y*)	(*X*) × (*Y*)
Tropical forests	7	17.3	17.5 × 10^12^	303 × 10^12^
Temperate and boreal forests	18	9.0	24.1 × 10^12^	217 × 10^12^
Tropical grasslands and savannas	11	4.1	27.6 × 10^12^	113 × 10^12^
Temperate grasslands and Mediterranean shrublands	8	3.6	17.8 × 10^12^	64 × 10^12^
Annual cropland	13	0.25	13.5 × 10^12^	3.4 × 10^12^
Deserts	3	0.6	27.7 × 10^12^	16.6 × 10^12^
Arctic tundra	5	0.7	5.6 × 10^12^	3.9 × 10^12^
Global total (without ice-covered area)			133.8 × 10^12^	721 × 10^12^

Computation of the prey kill values for each type of biome based on data (spider biomass m^−2^) from Table 1

- Assumption 1: Spiders have pulsed feeding patterns, with periods of excessive feeding (when food is very abundant) alternating with episodes of starvation (when prey gets scarce or spiders are inactive) (Turnbull 1973; Anderson 1974). During periods of high feeding activity, the spiders store surplus energy in their body’s interstitial tissue as lipid or glycogen (Foelix 2011). The spiders depend on these stored energy reserves during periods of starvation (e.g., on rainy days). To estimate the annual prey kill of the global spider community, we proceed from an overall mean food intake which is intermediate between pronounced high or low daily food consumption. After an extensive literature survey on spider feeding, we propose an average daily food ingestion rate of ≈0.1 mg per milligram spider body mass which is the equivalent to ≈10% of a spider’s body weight (all values expressed as fresh weight). A daily food intake in this order of magnitude appears to be typical of most species of free-living araneomorph spiders in forests, grasslands, and agroecosystems (see Edgar 1970; Robinson and Robinson 1970; Van Hook 1971; Foelix 2011; Nyffeler, pers. obs.) except a few rare cases of extraordinarily low food intake (e.g., Santana et al. 1990; Henschel 1997). In the case of desert spiders, a daily food ingestion rate of 0.01–0.04 mg per milligram spider body mass was used (see Lubin and Henschel 1996; Henschel 1997). Incidents of wasteful killing (and coupled with it “partial consumption”) in cursorial spiders apparently occur very rarely under natural conditions (Nyffeler, pers. obs.), and this issue has therefore not been taken into account in this study.

- Assumption 2: Spiders ingest, on average, ≈80% of the biomass of a killed prey (Edgar 1971; Moulder and Reichle 1972). Hence, we proceed with the assumption that the daily prey kill equals the daily amount of food ingested multiplied by the factor 1.25.

- Assumption 3: We assume that spiders forage on 365 days year^−1^ in tropical forests, on 240 days year^−1^ in deserts, on 180 days year^−1^ in temperate forests as well as tropical and temperate grasslands, on 120 days year^−1^ in the arctic tundra, and on 60–130 days year^−1^ in annual cropland (Kajak et al. 1971; Robinson and Robinson 1970, 1973, 1974; Breymeyer 1978; Shook 1978; Nyffeler 1982; Byzova et al. 1995). The contribution of winter-active spiders in terms of prey kill in temperate and cold climates (see Aitchison 1984) is considered to be very low and has therefore been neglected.

- Assumption 4: Spider biomass in forests has in most cases been assessed with the Berlese-Tullgren funnel method. This technique is limited to the investigation of spiders on the forest floor, and the calculated biomass values underestimate true biomasses. In temperate forests, at least 20% of the spider biomass are found in the canopy and understory (see Turnbull 1960; Reichle and Crossley 1967; Moulder and Reichle 1972; Zitnanska 1981). This pattern seems to hold for tropical forests (see Basset et al. 1992; Silva 1996; Yanoviak et al. 2003; Ellwood and Foster 2004; Dial et al. 2006). By multiplying the litter spider biomass values with a correction factor of 1.25, estimates for the total spider biomass in temperate, boreal, and tropical forests were obtained (Table 1).

- Assumption 5: For biomass m^−2^ of spiders associated with Mediterranean shrublands, no data are available. We arbitrarily placed this biome type in the category “Temperate grasslands (old fields, permanent pastures, mown meadows)”. The area size of Mediterranean shrublands is small (2.8 × 10^12^ m^2^) relative to the global terrestrial area, and a possible error resulting from insufficient data can be considered to be negligible.

*Method II* **:** The second approach is based on published studies of the annual prey kill of spider communities in various biome types (see Kirchner 1964; Reichle and Crossley 1967; Kajak et al. 1971; Van Hook 1971; Moulder and Reichle 1972; Robinson and Robinson 1974; Luczak 1975; Nyffeler 1976, 1982; Nyffeler and Benz 1978, 1979, 1988a,b, 1989; Schaefer 1990; Ysnel 1993; Jmhasly and Nentwig 1995; Malt 1996). For purposes of comparison, all prey kill values (including those expressed in terms of energy flow) were converted to grams of fresh weight m^−2^ year^−1^. Values were converted taking into account a prey water content of ≈75% (see Hagstrum 1970; Edgar 1971) and a caloric equivalent of prey of ≈23.5 kJ g^−1^ dry weight (mean value from literature data, see Hagstrum 1970; Moulder and Reichle 1972). Thus, 1 g fresh weight prey biomass equals ≈5.875 kJ. By means of extrapolation, the annual prey kill of the global spider community was computed, taking into account the global coverage of the different biome types. The global annual prey kill assessment with method II was based on the following assumptions:

- Assumption 1: Assessments of the annual prey kill of spider communities in tropical forests are currently unavailable. In lieu thereof, a study by Robinson and Robinson (1974) on the prey kill by the web-building spider community of an insecticide-free coffee plantation in New Guinea was used as a surrogate. We were operating on the assumption that the spider communities of tropical insecticide-free coffee plantations are to some degree comparable to those of tropical forests, given that coffee plantations are inhabited to a large extent by tropical woodland spiders (e.g., *Nephila maculata*) (Robinson and Robinson 1973, 1974; Robinson et al. 1974; Lubin 1978). Robinson and Robinson (1974) came to the conclusion that the web-building spider community in their study killed 16 g insect prey m^−2^ year^−1^. These authors suggested that the annual prey kill may even have been twice as high (≈32 g insect prey m^−2^ year^−1^) if hunting spiders would have been considered. The spider density in this coffee plantation (5.8 individuals m^−2^) was higher than the reported densities in the understory of tropical rain forests (3.3–3.6 individuals m^−2^; Rypstra 1986; Reagan and Waide 1996). However, we have to take into account that coffee plants reach a height of only 3–3.5 m, whereas tropical forest trees grow to a height of up to 55 m (Silva 1996). The canopy of tropical forests is inhabited by an abundant spider fauna (Basset et al. 1992; Russell-Smith and Stork 1994; Silva 1996; Ellwood and Foster 2004), and it is to be expected that those spiders kill considerable numbers of insects in addition to the insects killed by the spiders of the understory. Thus, it is well possible that the annual prey kill by spiders in tropical forests does exceed the conservative estimate of 16 g insect prey m^−2^ year^−1^.

- Assumption 2: Annual prey kill values for temperate forests appear to vary widely. Kirchner (1964) estimated an annual prey kill of ≈10 g m^−2^ year^−1^ for a semi-natural temperate forest in Central Europe, whereas lower values were reported for managed temperate forests. The annual prey kill in managed temperate deciduous forests in North America and Central Europe was estimated at ≈2 g m^−2^ year^−1^ (calculated by combining data for the spiders of the forest floor, understory, and canopy [Reichle and Crossley 1967; Moulder and Reichle 1972; Schaefer 1990]).

- Assumption 3: Annual prey kill values for unmanaged grasslands vary widely from ≈2 g m^−2^ year^−1^ (Ysnel 1993) up to >10 g m^−2^ year^−1^ (Kajak et al. 1971; Nyffeler and Benz 1989). In order to avoid overestimation, we used a conservative annual prey kill range of 2–10 g m^−2^ year^−1^ for grasslands and savannas (Table 3). The split in global coverage between unmanaged grassland/savannas (13.7 × 10^12^ m^2^) vs. permanent pastures/mown meadows (28.9 × 10^12^ m^2^) was based on SAGE/GTAP data http://www.agter.org/images/merlet_c2a_cultivablelands_G1.png

**Table 3:** Estimated annual prey kill by the global spider community (expressed as fresh weight g year^−1^) assessed with method II

Biome type	Number of assessments	Prey kill (g m^−2^ year^−1^)	Area in m^2^	Prey kill of entire area (g year^−1^)
		(*X*)	(*Y*)	(*X*) × (*Y*)
Tropical forests^a^	1	16	17.5 × 10^12^	280 × 10^12^
Temperate and boreal forests^b^	3	2–10	24.1 × 10^12^	48–240 × 10^12^
Unmanaged grasslands and savannas^c^	7	2–10	13.7 × 10^12^	27–137 × 10^12^
Permanent pastures and mown meadows/Mediterranean shrublands^d^	2	1–4	31.7 × 10^12^	32–127 × 10^12^
Annual cropland^e^	4	0.1–1	13.5 × 10^12^	1–14 × 10^12^
Others (urban areas, deserts, arctic tundra, etc.)^f^	1	0.2	33.3 × 10^12^	7 × 10^12^
Global total (without ice-covered area)			133.8 × 10^12^	395–805 × 10^12^

^a^Robinson and Robinson (1974)

^b^Kirchner (1964); combined data Reichle and Crossley (1967)/Moulder and Reichle (1972); Schaefer (1990)

^c^Bristowe (1958); Kajak et al. (1971); Van Hook (1971); Nyffeler and Benz (1978, 1989); Ysnel (1993); Malt (1996)

^d^Kajak et al. (1971); Nyffeler (1982)

^e^Luczak (1975); Nyffeler and Benz (1979); Nyffeler and Benz (1988a,b); Jmhasly and Nentwig (1995)

^f^Nyffeler (1976)

- Assumption 4: Published prey kill records for desert spider or arctic tundra communities are not available. Nevertheless, based on published natural history data (Polis 1991; Henschel 1997), we conclude that the annual prey kill in deserts is most likely very low. One might compare deserts to some degree to urban environments where the annual prey kill by spiders is also very low (0.2 g m^−2^ year^−1^; Nyffeler 1976). Supposing that the annual prey kill in deserts might be equally low as in urban areas, we arbitrarily assigned an assumptive value of ≈0.2 g m^−2^ year^−1^ to the spider communities in desert areas in order to be able to compute the global annual prey kill with method II. Due to many similarities between tundra and agricultural habitats (regarding population densities, body size composition and faunistic composition), we assume that the annual prey kill in arctic tundra sites might be comparable in magnitude to field crops in Europe (≈0.1–1 g m^−2^ year^−1^; Table 3).

Summing up the estimated prey kill subtotals for the seven biome types produced a second estimate of the annual prey kill of the global spider community.

## Results

Based on estimates of the average spider biomass m^−2^ in various terrestrial biomes, extrapolation suggests that the total standing biomass of the global spider community equals 25.09 × 10^12^ g (= 25 million metric tons fresh weight; Table 1). Biomass estimates m^−2^ follow the order forests > grasslands/shrublands > croplands, deserts, and tundra, which also reflects the order of total spider biomass per biome type worldwide.

The calculation of the annual prey kill by the global spider community with method I resulted in an estimate of 721 × 10^12^ g year^−1^ (= roughly 700 million tons year^−1^; Table 2). To derive an estimate to which degree reduced feeding activity during rainy days would affect the global annual prey kill, we recalculated this estimate assuming that it rained during one third of the feeding season with no prey being captured on rainy days. This simple scenario still leads to a global annual prey kill of 460 million tons year^−1^. Estimates derived from method I therefore suggest that the annual prey kill of the global spider community may be in the range of 460–700 million tons year^−1^. Our assessment with method II produced an estimate of the global annual prey kill of 395–805 × 10^12^ g year^−1^ (= roughly 400–800 million tons year^−1^; Table 3). The estimates computed with the two methods are highly comparable in magnitude (Tables 2 and 3). Together, the two different methods suggest that the global annual prey kill is presumably in the range of 400–800 million tons year^−1^.

## Discussion

Our extrapolations resulted in an estimated annual prey kill by the global spider community in the range of 400–800 million tons year^−1^ (Tables 2 and 3), which equals ≈1‰ of the global terrestrial net primary production (see Vitousek et al. 1986). For comparison, the human world population does consume an estimated 400 million tons of meat and fish annually (Bruinsma 2003; Food and Agriculture Organization of the United Nations 2014). Furthermore, our estimates for spiders appear to be of the same order of magnitude as the prey kill by whales (Cetacea) in the world’s oceans which has been estimated to be in the range of 280–500 million tons annually (Yodzis 2001). By contrast, the annual food consumption of all the world’s seabirds is estimated at 70 million tons (Brooke 2004).

### Which prey groups are killed by the spider global community?

Overall, fewer than 10 arthropod orders (Diptera, Hemiptera, Hymenoptera, Collembola, Coleoptera, Lepidoptera, Orthoptera, and Araneae) make up the majority of the prey of spiders (e.g., Turnbull 1960; Kajak et al. 1968, 1971; Robinson and Robinson 1970, 1973, 1974; Nyffeler 1982, 1999). Apart from insects and collembolans, spiders are another important component in spider diets (“intraguild predation” sensu Polis et al. 1989). Spider communities in the warmer areas (often dominated by cursorial hunters) have a higher percentage of spiders in their diets than spider communities in the colder climates (see Nyffeler 1999; Nyffeler and Sunderland 2003) and cursorial spiders feed more frequently on other spiders than web-builders (Birkhofer and Wolters 2012). Spiders feed on all stages (eggs, immatures, and adults) of their arthropod prey (Nyffeler et al. 1990). In addition to this, some larger spiders occasionally prey on earthworms, slugs, snails, and small vertebrates (Foelix 2011). Also, spiders have been reported supplementing their animal diet by feeding on plant materials (Nyffeler et al. 2016).

### Relative contribution of different biome categories to the global annual prey kill

Spiders in forests and grasslands accounted for more than 95% of the annual prey kill of the global spider community (i.e., 697 million tons year^−1^ estimated with method I and 387–784 million tons year^−1^ with method II), whereas spiders in other biomes (i.e., annual crop fields, urban areas, deserts, arctic tundra) were less significant contributors to the global prey kill (24 million tons year^−1^ estimated with method I and 8–21 million tons year^−1^ with method II, Tables 2 and 3). Forests, grasslands, and savannas cover an area of 87 million km^2^, which is about two thirds of the global terrestrial surface area (Saugier et al. 2001). Forests, grasslands, and savannas are less frequently disturbed than, e.g., agricultural or urban areas and allow spider populations to build up a higher biomass (Table 1). Both these differences explain the high prey kill in these biomes (Tables 2 and 3). These spiders further feed on many forest and grassland pest species underlining the important role of spiders as providers of biological control services (e.g., Juillet 1961; Kirchner 1964, 1967; Pointing 1966; Kajak et al. 1968, 1971; Van Hook 1971; Jennings and Pase 1975; Furuta 1977; Schmitz 1993; Oedekoven and Joern 1998). However, spiders do not only kill pest prey but also consume other beneficial arthropods (including large numbers of honey bees) in forest and grassland habitats (Nyffeler and Benz 1978; Malt 1996).

In relation to pest control, it is noteworthy that the spiders associated with annual crops only contribute less than 2% to the global annual prey kill. This can be explained by the fact that annual crop fields are “disturbed habitats” characterized by low spider biomass and a relatively short feeding season (Luczak 1979; Nyffeler and Benz 1979; Nyffeler et al. 1994a). Nevertheless, in wheat-, rice-, and cotton-growing areas with no or very low pesticide usage, the presence of spiders (in combination with other predators) may at times have a beneficial effect in slowing down the population growth of hemipteran pests (Kiritani et al. 1972; Sunderland et al. 1986; Nyffeler and Benz 1987, 1988a,b; Nyffeler et al. 1992, 1994a,b; Jmhasly and Nentwig 1995; Birkhofer et al. 2008, 2016).

Desert spiders only account for a small percentage (≤2%) of the global annual prey kill, but deserts cover a vast area (27.7 million km^2^) of the globe (Saugier et al. 2001). Due to adverse environmental conditions, prey availability in deserts is very low and these biomes are often populated by spiders in very low densities (Shook 1978; Polis 1991; Lubin and Henschel 1996; Henschel 1997). There are exceptions to this trend. Polis and Hurd (1995) described small desert islands in the Gulf of California, Mexico, where spiders occur in extraordinarily high numbers as a consequence of allochthonous energy input from the ocean. But the area of such island deserts is so small compared to the globe’s total desert area that its contribution to the global spider prey kill must be considered as negligible. Despite the fact that prey biomass killed m^−2^ by desert spiders appears to be relatively low compared to spider communities in other biomes, these arachnids are noticeable top predators in desert food webs (Polis and McCormick 1986; Polis 1991; Polis and Yamashita 1991). The relative contribution of spiders of the Arctic tundra to the global annual prey kill is equally low as for desert-living spider communities (<1%). The climatic seasonality and short foraging periods in this biome certainly contribute to those low estimates. However, spiders in the Arctic tundra (dominated by individuals from the family Linyphiidae) play an important ecological role by entrapping in their webs nutrients that originated from wind-blown allochthonous inputs (mostly chironomid midges). These nutrients are thereafter retained within the system and contribute to early stages of ecosystem development (Hodkinson et al. 2001).

### Experimental evidence supporting our estimates of the global annual prey kill

Our estimates of the global annual prey kill (Tables 2 and 3) imply that spiders exert considerable predation pressure on insect populations, especially in forests and grasslands. These estimates are supported by exclusion studies in forest and grassland habitats in different parts of the world (Clarke and Grant 1968; Kajak et al. 1968; Schmitz 1993; Oedekoven and Joern 1998; Lawrence and Wise 2000; Tanhuanpää et al. 2001). After spiders had been manually removed from experimental exclosure plots, a significant increase of the number of insects and collembolans was noticed compared to control plots with spiders. Greenstone (1978), however, stated that even though spiders can achieve very high densities and consume large quantities of insect prey, they may not necessarily have a significant role in the regulation of insect populations. Spiders depress herbivorous insect populations by 1–20% (Juillet 1961; Pointing 1966; Kirchner 1967; Furuta 1977; Nakamura and Nakamura 1977; Oedekoven and Joern 1998; Tanhuanpää et al. 2001). Acting in concert with other natural enemies (ants, ground beetles, predaceous bugs, birds, etc.), spider communities certainly exert significant predation pressure on herbivorous insect populations (e.g., Kajak et al. 1971).

### Further evidence in support of our theory of high global prey kill by spiders

There is also indirect support for the significance of spider predation in terrestrial biomes: a large number of insect species have evolved morphological and/or behavioral adaptations to reduce the risk of predation by spiders (see Eisner et al. 1964; Wise 1993). This concerns in particular the adults of ten thousands of species of moths, butterflies, and skippers (Lepidoptera) which evolved an escape mechanism allowing them to disentangle themselves from the webs of ecribellate spiders (see Eisner et al. 1964). Eisner et al. (1964) stated “….Moths, by virtue of the loose scales that cover their wings and bodies, are admirably adapted to elude capture by orb-weaving spiders. Rather than sticking to the web, these may simply lose some of their scales to the viscid threads, and then fly on.”

Further indirect evidence for the enormous ecological significance of spiders is that they serve as a food source for an incredibly diverse complex of arthropod-eating carnivores and, given our estimated global spider biomass (25 million metric tons fresh weight), spiders certainly are a crucial source of nutrition for many predator species. An estimated 8000–10,000 species of highly specialized predators/parasitoids/parasites feed exclusively on spiders, all of them being evolutionarily adapted to this special lifestyle (Table 4). In addition to this, an estimated 3000–5000 polyphagous bird species include spiders in their diets. Many of these bird species feed heavily on spiders in certain periods (i.e., spiders constituting 20–95% of the birds’ prey biomass [Kuitunen and Törmälä 1983; Poulin and Lefebvre 1996; Naef-Daenzer et al. 2000]). Likewise, many species of frogs, toads, salamanders, newts, lizards, snakes, shrews, mice, rats, and bats include spiders in their diets (Shine 1986; Schulz and Wainer 1997; Van Sluys and Rocha 1998; Marques et al. 2006). Even fish from more than 20 families have been reported consuming spiders that mistakenly landed on the water surface after ballooning across lakes or that fell into water from overhanging trees (e.g., Bristowe 1941; David and Closs 2003).

**Table 4:** Estimated global number of species of highly specialized predators, parasitoids, and parasites of spiders/spider eggs

Taxon	Estimated number of species	Hunting strategy	Source
Hymenoptera			
Pompilidae	≈5000 described; possibly ≥500 undescribed	Predator of spiders	Pitts et al. 2006; James Pitts, pers. comm.
Crabronidae (*Miscophus*, *Pison*, *Pisonopsis*, *Trypoxylon*)	≈1000	Predator of spiders	Pulawski 2016
Sphecidae (*Chalybion*, *Sceliphron*)	≈80	Predator of spiders	Pulawski 2016
Ichneumonidae (Polysphincta)	≈190	Spider ectoparasitoid	Gauld and Dubois 2006
Ichneumonidae (excluding Polysphincta)	Dozens	Egg sac parasitoid	Austin 1985; Fitton et al. 1987
Scelionidae (Baeines)	≈440 described; possibly ≈2500 undescribed	Egg parasitoid	Iqbal and Austin 2000; Johnson 2016; Lubomir Masner, pers. comm.
Encyrtidae (*Amira*)	Several	Egg parasite	Noyes 1977
Eulophidae (*Aranobroter*, *Aprostocetus*, *Baryscapus*, *Pediobius*, and others)	≈20 described; possibly ≈80 undescribed	Egg predator	LaSalle 1990; John LaSalle, pers. comm.
Eupelmidae (*Arachnophaga*)	Several	Egg predator	Muma and Stone 1971
Formicidae (*Proceratium*, *Discothyrea*)	Several	Egg predator	Brown 1980; Dejean et al. 1999
Pteromalidae (*Arachnopteromalus*)	Several	Egg predator	Peaslee and Peck 1983; Austin 1985
Diptera			
Acroceridae (*Acrocera*, *Ogcodes*, and others)	≈500	Spider endoparasitoid	Borkent and Schlinger 2008
Tachinidae	1	Spider endoparasitoid	Vincent 1985
Therevidae	1	External parasite	Ramírez 1995
Chloropidae (*Pseudogaurax*)	Several	Egg predator	Wheeler 2003
Drosophilidae (*Titanochaeta*)	11	Egg predator	O’Grady et al. 2003
Ephydridae (*Trimerina*)	1	Egg predator	Foote 1984
Phoridae (*Megaselia*)	Dozens	Egg predator/spider endoparasitoid	Disney 2008, 2012
Sarcophagidae (*Baranovisca*, *Mehria*)	22	Egg predator/parasitoid	Pape 1996; Thomas Pape, pers. comm.
Lepidoptera			
Cosmopterigidae (*Anatrachyntis*)	1	Egg predator	Austin 1985
Stathmopodidae (*Stathmopoda*)	1	Egg predator	Austin 1985
Neuroptera			
Mantispidae	334	Egg predator	Ohl 2004
Odonata			
Pseudostigmatidae	≈20	Predator of spiders	Ingley et al. 2012
Hemiptera			
Reduviidae (*Stenolemus*)	Several	Predator of spiders	Soley et al. 2011
Acari			
Erythraeidae / Laelapidae	Several	Parasite of spiders	Welbourn and Young 1988
Scorpiones			
Buthidae (*Isometroides*)	1	Predator of spiders	Main 1956
Nematodes			
Mermitidae	Possibly hundreds	Parasite of spiders	Poinar 1987; George Poinar, pers. comm.
Fungi—Hypocreales			
Cordycipitaceae (*Akanthomyces*, *Cordyceps*, *Gibellula*, *Torrubiella*) and others	≈50–100	Parasite of spiders	Evans 2013; Nyffeler et al. 2016
Total	≈8000–10,000		

### Nonlethal effects of spiders on prey additionally increase their global impact

In recent years, researchers have shown experimentally that spiders not only affect insects by inflicting mortality on them, they additionally have indirect (nonlethal) effects by intimidating them to the point where insects reduce their feeding activity in the presence of spiders in order to decrease predation risk (Schmitz et al. 1997; Schmitz 1998; Snyder and Wise 2000; Hlivko and Rypstra 2003). Such nonlethal effects of spiders can result in reduced herbivore damage. Hlivko and Rypstra (2003) noted “….the potential impact of spiders may be underestimated in food web studies that only consider predation rates….” Thus, the spiders’ huge predation impact documented in our study (Tables 2 and 3) is even further enhanced due to additional nonlethal effects.

## Concluding remarks

The estimated standing biomass of the global spider community is impressive (≈25 million tons, Table 1). There are few groups of terrestrial predaceous arthropods that can compare with spiders in terms of abundance and biomass. An exception are ants (Formicidae estimated at approx. 280 million tons, Hölldobler and Wilson 1994), but most species of ants are omnivorous and utilize a much broader range of food resources including considerable amounts of plant materials (Hölldobler and Wilson 1990). Here, we suggest that the predation impact of spider communities is particularly high in forest and grassland biomes, with considerably lower impact in desert, urban, and tundra biomes. These estimates emphasize the important role that spider predation plays in semi-natural and natural habitats, as many economically important pests and disease vectors breed in those forest and grassland biomes. We hope that these estimates and their significant magnitude raise public awareness and increase the level of appreciation for the important global role of spiders in terrestrial food webs.
